# The Chemical Evolution of the La_0.6_Sr_0.4_CoO_3−δ_ Surface Under SOFC Operating Conditions and Its Implications for Electrochemical Oxygen Exchange Activity

**DOI:** 10.1007/s11244-018-1068-1

**Published:** 2018-10-20

**Authors:** Alexander K. Opitz, Christoph Rameshan, Markus Kubicek, Ghislain M. Rupp, Andreas Nenning, Thomas Götsch, Raoul Blume, Michael Hävecker, Axel Knop-Gericke, Günther Rupprechter, Bernhard Klötzer, Jürgen Fleig

**Affiliations:** 1 Institute of Chemical Technologies and Analytics, Vienna University of Technology, Getreidemarkt 9/164-EC, 1060 Vienna, Austria; 2 Institute of Materials Chemistry, Vienna University of Technology, Getreidemarkt 9/165-PC, 1060 Vienna, Austria; 3 Institute of Physical Chemistry, University of Innsbruck, Innrain 52c, 6020 Innsbruck, Austria; 4 Department of Inorganic Chemistry, Fritz Haber Institute of the Max Planck Society, Faradayweg 4-6, 14195 Berlin, Germany; 5 Department of Heterogeneous Reactions, Max-Planck-Institute for Chemical Energy Conversion, Stiftstraße 34-36, 45413 Mülheim, Germany; 6 Present Address: Department of Materials Science and Engineering, MIT, 77 Massachusetts Avenue, 02139 Cambridge, MA USA

**Keywords:** Oxygen reduction, SOFC cathode, Strontium segregation, NAP-XPS, Impedance spectroscopy, Perovskite-type electrode

## Abstract

Owing to its extraordinary high activity for catalysing the oxygen exchange reaction, strontium doped LaCoO_3_ (LSC) is one of the most promising materials for solid oxide fuel cell (SOFC) cathodes. However, under SOFC operating conditions this material suffers from performance degradation. This loss of electrochemical activity has been extensively studied in the past and an accumulation of strontium at the LSC surface has been shown to be responsible for most of the degradation effects. The present study sheds further light onto LSC surface changes also occurring under SOFC operating conditions. In-situ near ambient pressure X-ray photoelectron spectroscopy measurements were conducted at temperatures between 400 and 790 °C. Simultaneously, electrochemical impedance measurements were performed to characterise the catalytic activity of the LSC electrode surface for O_2_ reduction. This combination allowed a correlation of the loss in electro-catalytic activity with the appearance of an additional La-containing Sr-oxide species at the LSC surface. This additional Sr-oxide species preferentially covers electrochemically active Co sites at the surface, and thus very effectively decreases the oxygen exchange performance of LSC. Formation of precipitates, in contrast, was found to play a less important role for the electrochemical degradation of LSC.

**Electronic supplementary material:**

The online version of this article (10.1007/s11244-018-1068-1) contains supplementary material, which is available to authorized users.

## Introduction

Solid oxide fuel cells (SOFCs) are highly efficient devices to convert chemically bound energy into electricity, which potentially makes them one of the future key technologies for a more environmental friendly energy production [1–3]. Nowadays, SOFCs are already commercially available, but their relatively high price still hampers a widespread breakthrough. A main reason for the high production costs are high operating temperatures of 700–900 °C, which prevent the use of cheaper materials for interconnectors and housing and, moreover, make SOFCs still prone to long term degradation [4–6]. To overcome these problems and to further increase the thermodynamic efficiency, a temperature reduction to 450–650 °C would be highly desirable.

When operating SOFCs at lower temperatures, especially the oxygen reduction reaction strongly contributes to the electrical losses—it is written here in Kröger-Vink notation, with O_O_^×^, V_O_^••^, and e^|^ denoting regular lattice oxygen, oxygen vacancy, and electronic point defect, respectively:

1 $${{\text{O}}_{\text{2}}}+{\text{ 2 }}{{\text{V}}_{\text{O}}}^{{ \bullet \bullet }}+{\text{ 4 }}{{\text{e}}^|} \rightleftarrows {\text{2 }}{{\text{O}}_{\text{O}}}^{ \times }.$$

Thus, the performance of such intermediate temperature SOFCs depends crucially on the electro-catalytic activity of the used cathode material. Mixed ionic and electronic conducting perovskite-type oxides such as (La,Sr)CoO_3−δ_ can exhibit very high activity for catalysing O_2_ reduction and have thus been extensively investigated within the past years [7–12]. Under controlled conditions a very low surface related resistance smaller than 0.5 Ωcm^2^ has been realised on Sr- as well as Ba-doped LaCoO_3_ (LSC) model-type thin film electrodes at 600 °C [13]. However, at the elevated temperatures of SOFC operation these materials are prone to comparatively strong degradation, which is often attributed to a segregation of the Sr dopant to the electrode surface [14–22]. There it seems to selectively block the electrochemically most active sites, which are probably related to cobalt at surface defects [23]. As a reason for this Sr segregation, the enhanced concentration of oxygen vacancies in the near-surface region of LSC is discussed. Owing to the electrostatic attraction between the relative positive charge of oxygen vacancies (denoted as V_O_^••^ in Kröger-Vink notation) and Sr as the relative negative dopant (Sr_La_^|^), the latter tends to diffuse towards the surface, where it accumulates [16].

While this model successfully describes the observed trend of Sr accumulation at the surface of LSC and related compounds such as (La,Sr) (Co,Fe)O_3−δ_ (LSCF), it does not specify in which chemical state or compound Sr finally resides at the surface. This topic is also controversially discussed in literature, with some studies observing only SrO termination layers on top of LSC and LSCF while others mention the formation of precipitates [17–22, 24–26]. Moreover, the detailed connection of Sr surface segregation and degradation of electrochemical activity are still under debate. For example, electrochemically already degraded LSC cathodes showing a predominantly SrO-terminated surface still undergo an increase of the oxygen exchange resistance even though their surface composition does not show any further measureable changes as demonstrated by low energy ion scattering (LEIS) [15]. Other studies either report an enhancement [27] or a decrease [23] of the electrode activity by SrO when being deposited on the electrode surface by pulsed laser deposition. One reason for these discrepancies may be related to the fact that most of the surface studies on LSC were conducted ex-situ in the past. Thus, the chemical state of the LSC surface during electrochemical characterization at elevated temperature may differ strongly from the analysed one.

A method to overcome this “ex-situ gap” and to study the chemical evolution of the LSC surface during electrochemical experiments at elevated temperatures is near ambient pressure X-ray photoelectron spectroscopy (NAP-XPS), which was already successfully employed to study the surface of various perovskite-type electrodes [28–32]. Besides the possibility of analysing the electrode surface close to SOFC operating conditions, the strength of this method lies in the identification of different chemical states of an element. NAP-XPS can thus yield a more detailed insight into surface chemistry than measuring only the surface concentration of segregated Sr.

In this study the evolution of the surface composition of La_0.6_Sr_0.4_CoO_3−δ_ electrodes was characterised by NAP-XPS at elevated temperatures while simultaneously measuring the surface activity of the material by means of impedance spectroscopy. Impedance spectroscopy is an electrochemical AC method, which provides a rather direct access to the electro-catalytic activity of an electrode. In the present case, the polarization resistance of the characterised LSC electrodes is inversely proportional to the equilibrium exchange rate of the oxygen exchange reaction—cf. Equation 1—which takes place on the electrode surface. By means of NAP-XPS the surface composition of LSC was monitored in-situ and a special focus was laid on the evolution of Sr and O species as a result of different applied temperatures between 400 and 780 °C. By these experiments, surface accumulation of strontium during annealing, as already suggested in other studies, could be confirmed [16, 30, 33]. Simultaneously, a degradation of the catalytic activity of LSC for the O_2_ reduction reaction was observed. More precisely, the decrease of electro-catalytic activity was accompanied by the formation of an additional Sr-oxide species appearing in the XPS data upon increasing the temperature. This third Sr species on the LSC surface may have already been observed in previous XPS studies but was never discussed in detail so far. Therefore, this paper focuses on a critical discussion of XPS data analysis with the goal of successfully separating the contributions of different Sr surface species and clarifying their role for the oxygen exchange kinetics of LSC thin film electrodes. The evolution of the new Sr-oxide species is interpreted in terms of an insulating SrO-rich phase, which selectively blocks Co sites and thus hampers the electrochemical surface activity for the oxygen exchange reaction.

## Experimental

### Sample Design and Preparation

The investigated La_0.6_Sr_0.4_CoO_3−δ_ (LSC) thin film electrodes were deposited by pulsed laser deposition (PLD) on both sides of yttria-stabilised zirconia (YSZ) single crystals. The entire sample preparation was carried out as follows: The LSC target was prepared by a modified Pechini method [34]. The starting materials La_2_O_3_, SrCO_3_, and metallic Co (all purchased from Sigma–Aldrich, 99.995% trace metals pure or higher) were separately dissolved in HNO_3_ (Sigma Aldrich, redistilled, 99.999% trace metals pure). The nitrate solutions were mixed in appropriate ratios and citric acid (Fluka, 99.9998% trace metals pure) was added in a molar ratio of 1.2 with respect to the cations. From the obtained solution water was evaporated by heating on a hot plate until a highly viscous foam was formed. Upon further heating, this foam decomposed spontaneously. The obtained powder was calcined at 1000 °C and cold-isostatically pressed into a pellet (ca. 1.5 kbar), which was subsequently sintered at 1200 °C for 12 h in air.

From this target thin films were deposited on both sides of a polished, (100)-oriented YSZ single crystal by PLD. Ablation of the material was done by a KrF excimer laser (Coherent Lambda COMPexPro 201F, 248 nm wavelength) with a pulse frequency of 5 Hz and an energy density on the target of ca. 1–1.5 J/cm^2^. The O_2_ background pressure in the PLD chamber was set to 4 × 10^−2^ mbar and the substrate was heated to 600 °C surface temperature (controlled by a pyrometer). Layers of approximately 200 nm were produced by applying 6750 laser pulses to the LSC target. After deposition the sample was cooled at a rate of 12°C/min in the deposition atmosphere. This procedure was repeated on the back side of the double side polished YSZ crystals for obtaining symmetrical samples.

Before performing spectroscopic studies on the LSC electrodes they were treated in double distilled water for 10 min to remove water soluble SrO-like surface layers, which may likely form already during the PLD process (since especially the firstly deposited thin film already underwent a heat treatment during deposition of the second electrode) [15].

### Near-Ambient Pressure XPS and Impedance Measurements

**Fig. 1:**
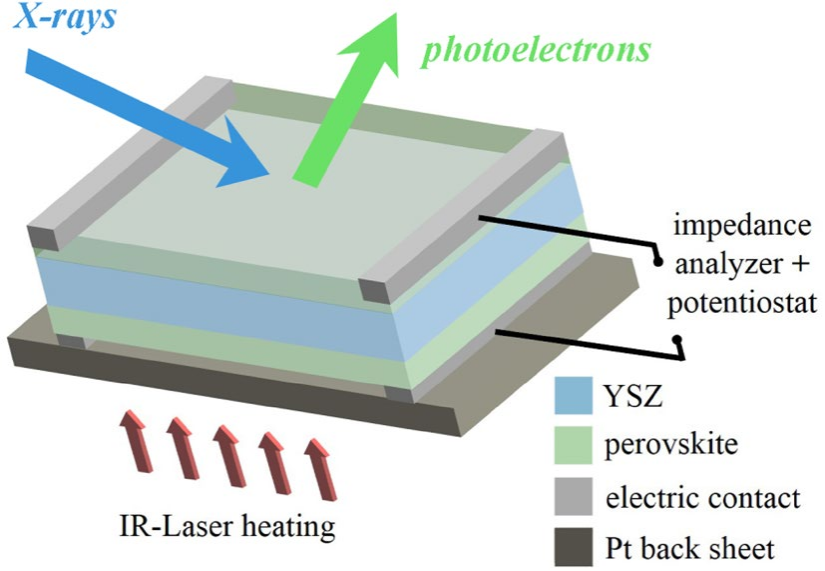
Sketch of the sample design for the in-situ experiments. It allows simultaneous NAP-XPS and electrochemical impedance measurements. The LSC electrodes are applied on both sides of an YSZ (100) single crystal. The sample was mounted by means of Pt/Ir clamps pressing on the top side of the sample. For improving the electrical contact, small stripes of Pt paste were applied between clamps and LSC at the edges of the sample

In-situ experiments were performed at the ISISS beam line of the HZB/BESSY II synchrotron in Berlin with a near-ambient pressure high energy X-ray photoelectron spectroscopy (XPS) setup, which enables measurements at elevated pressures (up to 7 mbar) with photon energies ranging from 80 to 2000 eV. The main parts are a “high pressure” chamber with an attached differentially pumped hemispherical analyser (modified SPECS Phoibos 150) including a 2D delay line detector. A detailed description of the near-ambient pressure XPS-setup is given in Ref [35]. A sketch of the sample holder, which was used for in-situ XPS experiments under electrochemical polarization and simultaneous impedance measurements, is shown in Fig. 1. The spectroscopically investigated electrode was connected to the grounded contact of the impedance analyser/potentiostat, the back side electrode to its positive pin. This was done to avoid possible XPS peak broadening due to the applied AC voltage for impedance measurements. Samples were heated via the platinum back sheet using an infrared laser. The temperature was monitored with a pyrometer measuring the electrode surface temperature, as well as by the conductivity of the YSZ electrolyte obtained from electrochemical impedance measurements [36, 37]. Both methods consistently yielded temperatures with a maximum deviation of ± 15 °C. In the following, the temperature values obtained by pyrometer are used, unless otherwise specified.

Electrochemical impedance measurements were carried out by an Alpha-A High Performance Frequency Analyser equipped with a POT/GAL 30V/2A interface (both: Novocontrol, Germany). Impedance spectroscopy was performed in a frequency range typically between 10 mHz and 1 MHz (but frequency was adjusted if necessary). The AC root mean square voltage was 5 mV.

The in-situ spectroscopic measurements were conducted in the following manner: After XPS characterisation of the electrode’s virgin, water-treated surface in UHV, 0.5 mbar O_2_ was introduced into the NAP-XPS chamber. While increasing the temperature stepwise up to 780 °C, XPS spectra were recorded in-situ and electrochemical impedance spectroscopy measurements were performed simultaneously. This allowed correlating surface chemical information with the electro-catalytic activity of the LSC electrodes.

Surface sensitive in-situ XPS spectra were obtained with different photon energies (252 eV for Sr 3d, 410 eV for C 1 s, 650 eV for O 1 s, 920 eV for Co 2p, and 975 eV for La 3d). These correspond to a narrow range of kinetic photoelectron energies between 110 and 130 eV, which lead to optimised surface sensitivity and an almost equal information depth (inelastic mean free path, IMFP) of 0.5–0.6 nm, according to the NIST Standard Reference Database [38]. For comparison, the lattice constant of cubic LSC is 0.38 nm [13]. In case of depth profiling measurements, the photon energies were increased in multiple steps, resulting in photoelectron energies of up to 730 eV corresponding to a maximum information depth (IMFP) of 1.5 nm. After each change of excitation energy a spectrum of the Fermi edge was recorded for calibration of the binding energy axis. All spectra were therefore referenced to the Fermi edge, to compensate for errors from monochromator mechanics at the synchrotron beamline [39].

## Results

### Parameterization of Impedance Data

**Fig. 2:**
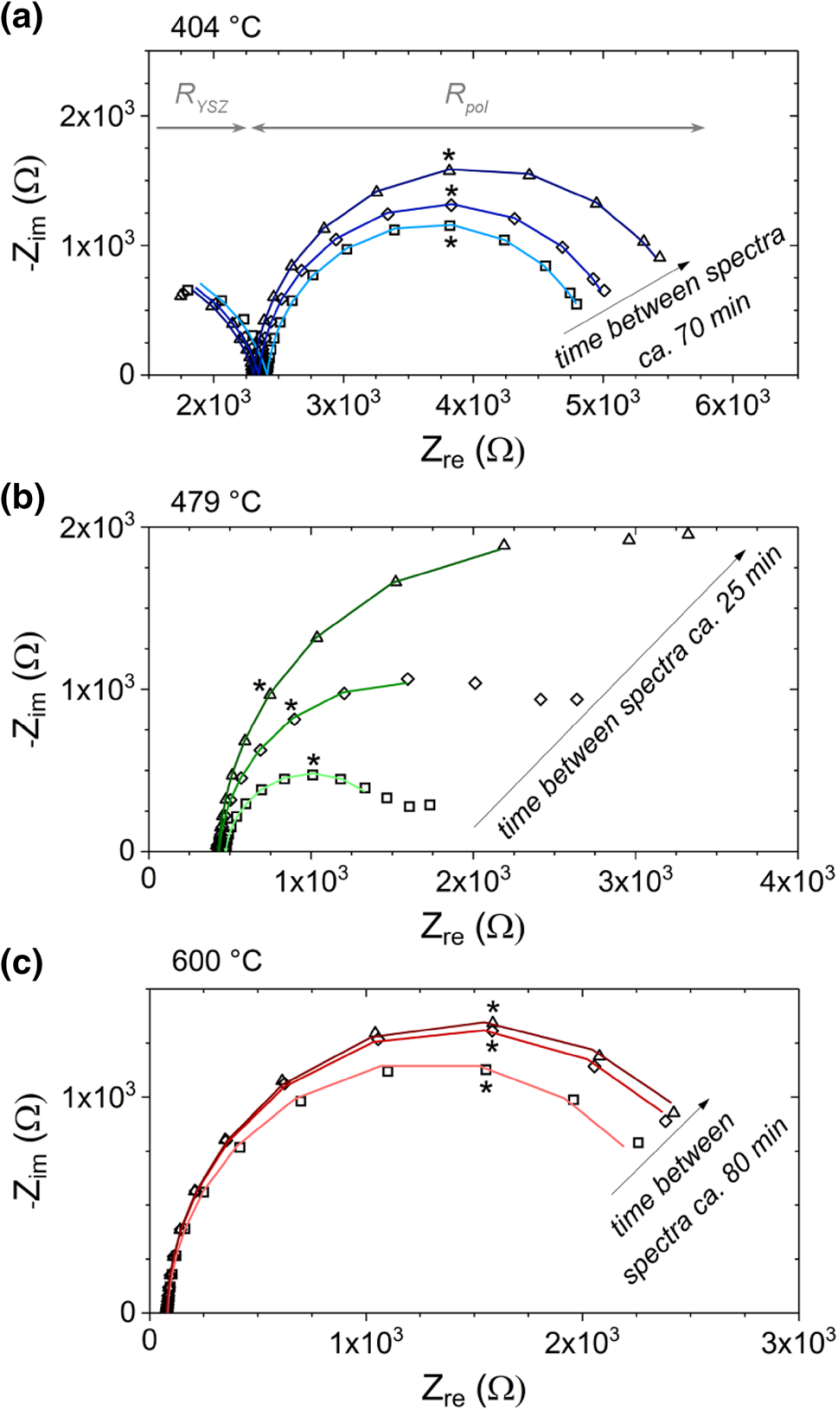
Impedance spectra (Nyquist plots) measured at different temperatures. Measured data are given by symbols, whereas fit results are shown by lines. The chronological sequence of the individual spectra is indicated by arrows. **a** Spectra recorded at ca. 400 °C; the asterisk marks the 40 mHz point. **b** 479 °C; the asterisk marks the 60 mHz point. **c** 600 °C; the asterisk marks the 12 mHz point

**Fig. 3:**
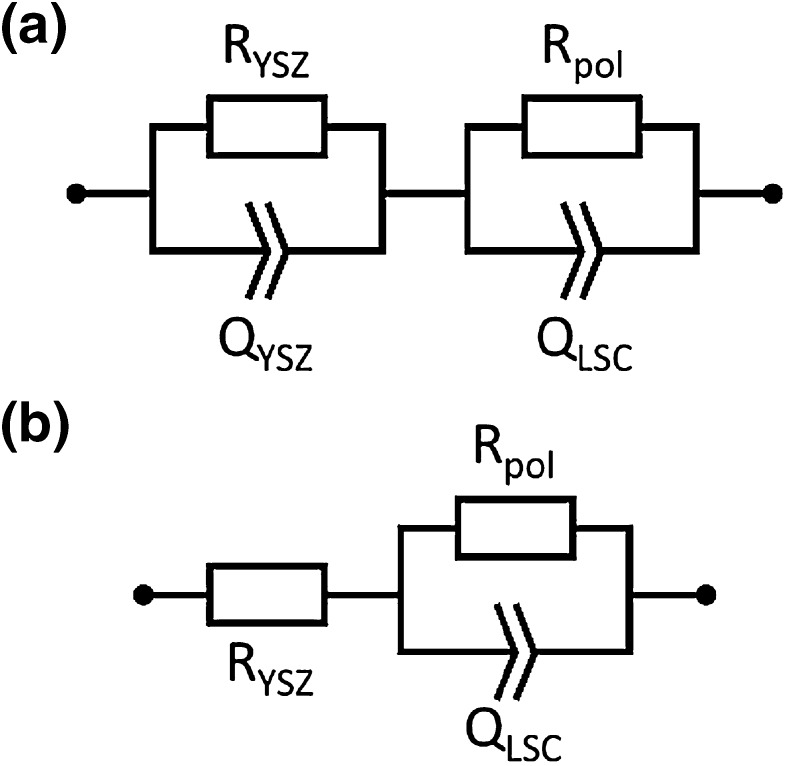
Equivalent circuits used for parameterization of impedance data measured at lower temperatures with partly resolved electrolyte arc (**a**) and for spectra at higher temperature exhibiting only a high frequency axis intercept (**b**)

Impedance spectra recorded at different temperatures are depicted in Fig. 2. Despite the temperature variation and ongoing degradation, all spectra consist of the same main contributions—a high frequency feature, which can be attributed to the electrolyte [40, 41] and a low frequency arc of the electrode [13]. The electrolyte feature appeared either as part of an arc at lower temperatures (see Fig. 2a) or as high frequency intercept with the real axis (see Fig. 2b, c). In data parameterization this fact was taken into account by adjusting the corresponding equivalent circuit. The equivalent circuits used for fitting of low and high temperature spectra are shown in Fig. 3a, b, respectively. In case of low temperature data the electrolyte arc is modelled by an RQ element (Fig. 3a), with R_YSZ_ denoting the resistance of ion conduction in YSZ and the constant phase element Q_YSZ_ representing a non-ideal capacitor [42]. For high temperature data with only a high frequency intercept the electrolyte contribution is fitted by the resistor R_YSZ_ (cf. Fig. 3b).

The electrode feature virtually consists of one slightly depressed semicircle, which is modelled by one RQ element for all experimental temperatures: Q_LSC_ is a non-ideal capacitor mainly representing the chemical capacitance of LSC [43, 44]. R_pol_ denotes the polarisation resistance of both LSC electrodes (working electrode at the front and counter electrode at the back side), which under the applied oxygen partial pressure of 0.5 mbar almost exclusively originates from the oxygen exchange process (cf. Eq. 1) at the surface [13]. The polarisation resistance is inversely proportional to the equilibrium reaction rate of the oxygen exchange reaction in Eq. 1. Thus, 1/R_pol_ is a direct measure for the catalytic activity of the LSC electrode surface for O_2_ reduction—i.e., an increase of R_pol_ reflects a decrease in the electro-catalytic activity. In the following discussion the main attention is drawn on the evolution of the polarisation resistance of LSC (R_pol_) upon heating. Its correlation with the defect chemistry measured by NAP-XPS will be discussed.

### XPS-Data Analysis

**Fig. 4:**
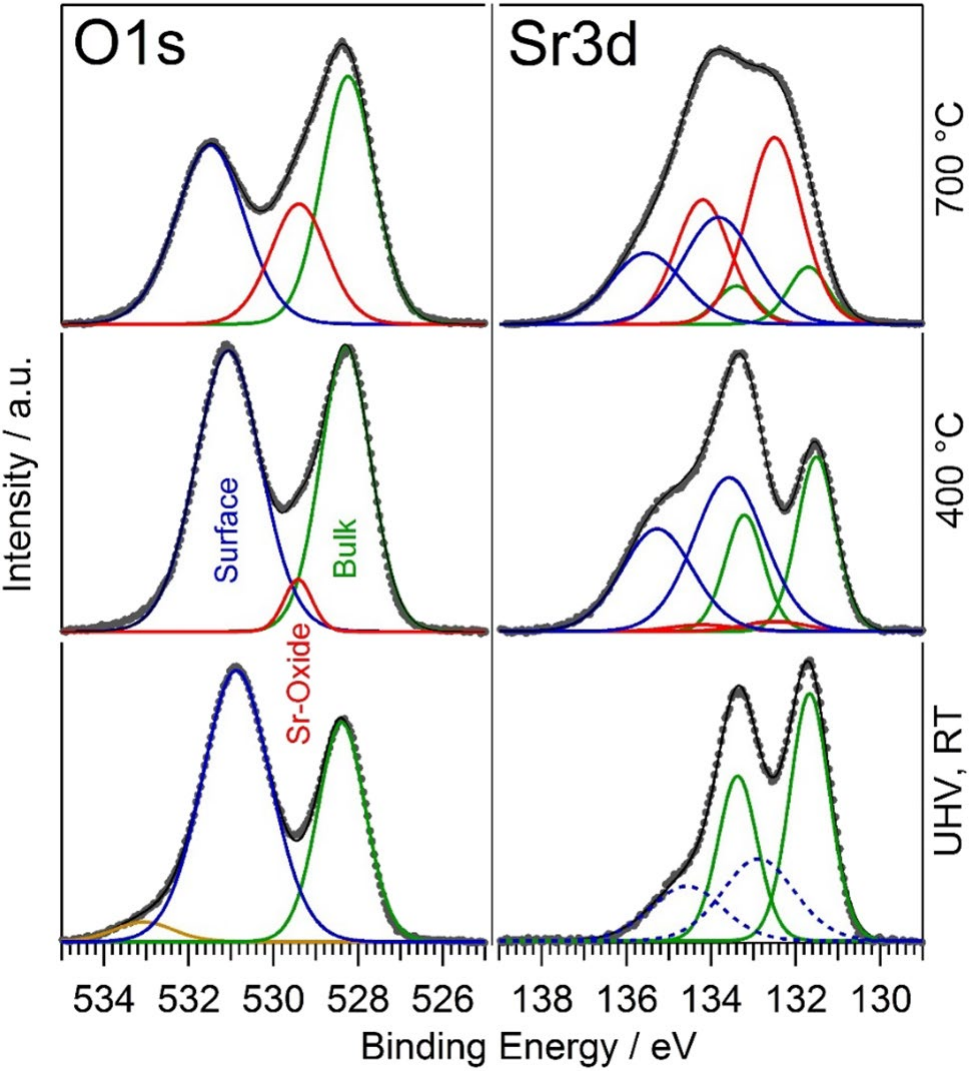
XPS spectra including peak fitting for the LSC electrode surface. The bottom spectra show the surface state of the virgin, water-treated sample directly after introduction into the UHV system at room temperature. Since before any heating the Sr3d species at higher BE may be affected by surface hydroxyls or adventitious carbon the fit here is shown with a dashed line; a corresponding C1s spectrum is shown in the supporting info (cf. Fig. S4). In the middle panels the initial state at 400 °C in 0.5 mbar O_2_ is shown and the top curves display a strongly degraded state after heating in 0.5 mbar O_2_ at 700 °C. For both O 1 s and Sr 3d the green component corresponds to the perovskite bulk signal of LSC. The blue component represents the surface signal of the perovskite. The additional red peaks of the degraded state result from the formation of a third Sr-oxide compound on the surface. Before heating also surface hydroxyls were observed—see component at ca. 533 eV in O 1 s spectra before heating (yellow component in bottom, left panel)

The spectra were fitted with CasaXPS, using a Shirley background subtraction and mixed Gaussian–Lorentzian (GL(30)) peak shapes for the Sr, Co, La and O components. The Sr 3d region was fitted with doublets, restricted by equal FWHM, fixed doublet separation of 1.7 eV (spin orbit splitting) and an area ratio of 2:3 [18]. For an appropriate fit of the Sr 3d spectra at least two different species had to be included in the fitting routine. For the sake of simplicity the high and low binding energy species are denoted as surface and bulk species, respectively [29, 30, 33]. A more detailed discussion of the interpretation of the different species is given in Sect. 4.1. Spectra collected on a virgin, water-treated sample before any heating could be completely explained by two contributions—see Fig. 4 (right hand side) and Fig. S1. Upon heating to 400 °C especially the Sr3d high BE species shifted to higher energies by about 1 eV, but a two component fit was still possible. (Further discussion regarding the justification of a three-component versus a two-component fit is given below.) The fit parameters such as position and full width at half maximum (FWHM) for surface and bulk component obtained on the spectrum measured directly after the very first heating step were used to constrain the peak positions with a tolerance of ± 0.3 eV in the fitting procedure of spectra collected on heat treated samples. Similarly, the O 1 s signal was fitted with single GL(30)-peaks for perovskite “bulk” and “surface” signal in case of the virgin sample—see Fig. 4 (left hand side). Also here the fit parameters obtained on the spectrum measured directly after the very first heating step served as a basis for constraining the parameters for the fits of XPS data from degraded LSC.

For both Sr 3d and O 1 s spectra a third species was introduced to account for the fact that especially for samples subjected to higher temperatures the measured data could not be satisfactorily explained with only two species. A detailed comparison of a two-species fit with a model considering three contributions for both Sr 3d and O 1 s is given in the supporting information—cf. Figs. S1 and S2, respectively. From this comparison—and thereby especially from O 1 s data—a three-species fit (i.e., considering bulk, surface and segregated Sr-oxide species) represents a more reasonable model for data collected at ca. 400 °C and above, and was thus applied for data interpretation throughout this paper. We would like to point out that we are well aware that for the samples at lowest temperatures this fitting approach may yield somewhat too high values for the third Sr species (with intermediate binding energy). However, to avoid any discontinuities in data representation we consistently applied one fitting model—assuming three Sr 3d and O 1 s species—to the entire data set.

The Co 2p signal was fitted with doublets restricted by equal FWHM, fixed doublet separation of 15.1 eV and area ratio of 1:2 [45]. For the Co 2p satellite feature an unconstrained single peak was used. The La 3d signal was fitted with two doublets for the La 3d main peaks and their strong satellites, according to Refs [46, 47]. Each La-doublet was restricted by equal FWHM, fixed doublet separation of 16.9 eV and an area ratio of 2:3.

For the calculation of the electrode surface composition the respective peak areas were corrected by cross sections for O 1 s, Sr 3d, La 3d and Co 2p (Elettra Trieste database, see Ref. [48]) and the energy-dependent photon flux characteristics of the beamline.

### Electron Microscopy

**Fig. 5:**
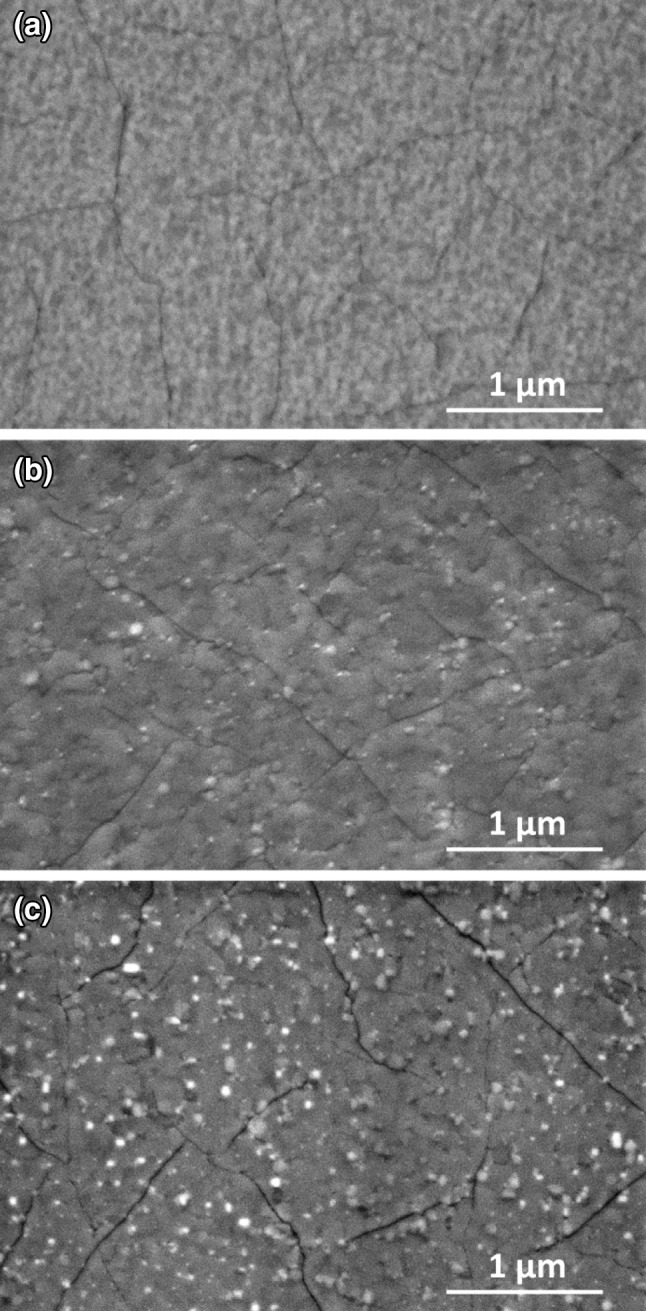
SEM images of the LSC electrodes at different stages of the combined NAP-XPS and electrochemical experiment. **a** Virgin, water-treated sample without any temperature treatments. **b** LSC surface after heating to 700 °C (sample no. LSC02). **c** LSC surface after heating to 790 °C (sample no. LSC01)

Scanning electron microscopy images—recorded with secondary electron contrast mode— are depicted in Fig. 5. Before any temperature treatment, the LSC films exhibit a slightly granular structure (see Fig. 5a), which can be assigned to their columnar growth on YSZ (100). This is in accordance with previously prepared LSC thin film electrodes [13, 15, 26]. Moreover, also the appearance of some cracks was already observed on the system LSC film/YSZ substrate. They form upon cooling after PLD deposition and are caused by the strongly different thermal expansion coefficient of the LSC thin film and the YSZ substrate. By heating to 700 °C a roughening of the LSC surface occurs (see Fig. 5b). Upon a further temperature increase to 790 °C some additional precipitates form on the surface—see the white spots in Fig. 5c. Formation of Sr-enriched particles on the surface of LSC triggered by annealing was also already reported in literature [19–21, 49]. A relationship of this particle formation and the evolution of LSC surface chemistry will be discussed below.

## Discussion

### Interpretation of Different Species in O 1 s and Sr 3d Spectra

As mentioned in Sect. 3.2 and shown in Fig. 4 both O 1 s and Sr 3d spectra can be explained by up to three components, depending on the degree of degradation of the surface. On virgin, water-treated LSC thin films before any heat treatment, two perovskite components were sufficient to satisfactorily describe the measured O 1 s and Sr 3d spectra—see Fig. 4, bottom panels. The lowest binding energy species in each case (O 1s: BE ≈ 528 eV, Sr 3d: BE ≈ 131.5 eV) is attributed to the perovskite bulk, which is in accordance with literature [29, 30, 33] as well as with our depth profiling measurements (see Fig. S3 in the supporting information). The interpretation of the surface species, with the highest binding energies (O 1s: BE ≈ 531.5 eV, Sr 3d: BE ≈ 133.5 eV) is less straightforward. In some studies it is assigned to the formation of SrO precipitates at the LSC surface [29, 30]. However, also surface SrCO_3_, Sr(OH)_2_ as well as an A-O-type perovskite termination layer are discussed as origin (A and B denote the different types of cations in the perovskite with general formula ABO_3_) [33].

In the present case, we can clearly rule out SrCO_3_ as the corresponding species, since no carbonate signal was found in C 1 s spectra (see Fig. S4). A small shoulder can be found in O 1 s spectra at ca. 533 eV on a virgin water-treated sample surface before any heating, which most probably originates from surface hydroxyls (see Fig. 4, bottom left panel). (Please note that for the virgin sample before any heating the presence of surface Sr(OH)_2_ may be responsible for the somewhat lower binding energy of the Sr “surface” component.) However, the surface hydroxyls completely vanished upon heating to ca. 400 °C, while the 531.5 eV feature in O 1 s and the 133.5 eV in Sr 3d spectra increased in relative intensity. This behaviour also rules out Sr(OH)_2_ as the origin of the high binding energy species. Since Sr 3d and O 1 s are the only elements, which show a clear ratio change between their two respective species in depth profiling measurements (see Fig. S3), the high binding energy surface species is suggested to be under-coordinated Sr and O on the surface of the perovskite. What also supports this interpretation is that a heat treatment of the LSC electrodes led to a significant change in the ratio of the Sr bulk and surface species, while the La 3d and Co 2p signals were virtually unaffected (compare Fig. 4 and S5).

Upon the first moderate heating to ca. 400 °C a significant change of the O 1 s and Sr 3d spectra was observed (see Fig. 4, middle panels). However, while keeping the temperature constant, the surface did virtually not show any further changes, which is depicted in Fig. S6 in the supporting info. Since the cation mobility in LSC at this temperature is too low for long range transport, this step-like change of the LSC surface during the first heating may be due to a surface reconstruction process. In addition, the observed changes may to some extent originate from the decomposition of surface species formed in ambient air such as the above mentioned surface hydroxyls or adventitious carbon (see Fig. S4). Therefore, an unambiguous quantification of the change of individual Sr species during this very first heating step is rather challenging.

Upon further increasing the temperature, significant changes in O 1 s and Sr 3d spectra could be observed (see Fig. 4, top panel) with the third Sr-oxide species strongly increasing in intensity. The average peak positions of the three different species in O 1 s and Sr 3d spectra are summarised in Table 1.

**Table 1:** Average XPS peak positions of the three different species obtained from fitting Sr 3d and O 1 s spectra

Species	Sr 3d 5/2 [eV]	O 1 s [eV]
Perovskite bulk	131.5 ± 0.1	528.2 ± 0.1
Perovskite surface	133.6 ± 0.2	531.3 ± 0.3
Third Sr-oxide species	132.9 ± 0.1	529.4 ± 0.1

### Evolution of Surface Chemistry and Electrochemical Activity upon Heating

**Fig. 6:**
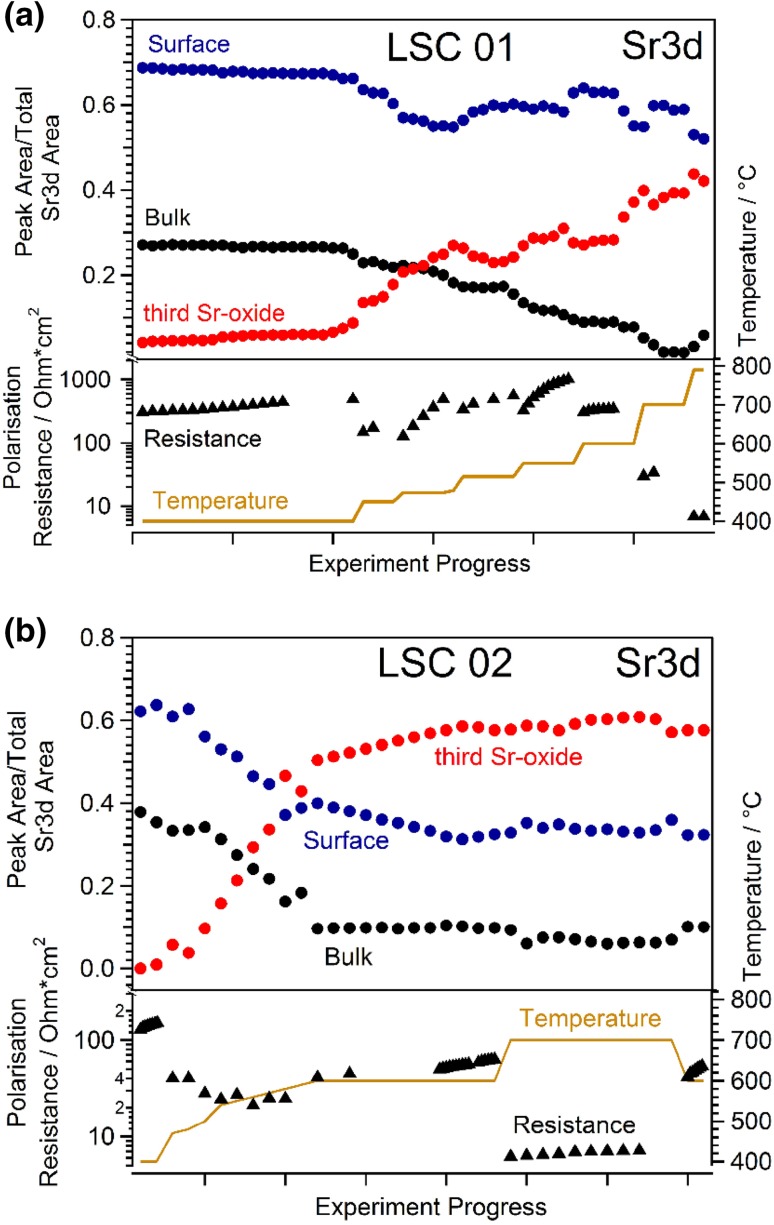
Evolution of the three Sr 3d species as well as the electrode polarization resistance upon increasing temperature in 0.5 mbar O_2_. Plots (**a**) and (**b**) show results on two different samples with slightly different heating profiles. The top panel in each case shows the evolution of the relative intensity of Sr 3d XPS peak areas with increasing reaction temperature. The respective peak areas for the perovskite surface (blue) and bulk species (black) and the third Sr-oxide species (red) were obtained from the fitting of the Sr 3d peak as shown in Fig. 4. The bottom panel in each case depicts the evolution of the total polarisation resistance of the LSC electrodes obtained by impedance spectroscopy

Figure 6 displays and correlates the results for the Sr 3d core level components and the impedance measurements for the in-situ experiment while heating two different LSC samples in 0.5 mbar O_2_ stepwise from ca. 400 °C to elevated temperatures. Please note that the Sr 3d peak area ratios in Fig. 6 as well as the polarisation resistance values are not plotted as a function of time, but rather versus the measurement number. This was done for the sake of a clearer representation of the overall trend, since the times to adjust different temperatures were quite different for practical reasons. However, since recording of XPS-spectra took approximately the same time during periods of constant temperature, this way of data representation still shows a qualitatively similar trend as a plot versus time, excluding the heating ramps. Moreover, degradation rates of the surface resistance are calculated for selected temperatures, for details see discussion.

For both LSC surfaces shown in Fig. 6, the observed trends in the evolution of the three Sr species are comparable. At ca. 400 °C only tiny amounts (if any) of the third Sr-oxide species can be detected and the strongest contribution comes from the species denoted as perovskite surface. This high surface sensitivity can be explained by the short IMFP of 0.5–0.6 nm of photoelectrons measured at the selected kinetic energy and is a distinct advantage of synchrotron-based XPS. Upon increasing the temperature above ca. 450 °C, however, the signal from the third Sr-oxide species strongly increases. At approximately 600 °C, its intensity has already reached the 8–11 fold value compared to ca. 400 °C. This increase of the third species is accompanied by a strong decrease of the perovskite bulk signal and a much weaker decrease of the perovskite surface contribution. The bulk signal dropped by a factor of 3–4 while the surface signal decreased by only 20–50%. This behaviour is a strong indication for the third Sr species to grow on top of the species denoted “perovskite surface”, which is believed to represent the reconstructed perovskite surface. Interestingly, keeping the sample at a temperature of 600–700 °C caused only comparably small changes despite the mobility of Sr in LSC is highly temperature dependent [50, 51] and it appears that the surface of LSC has already reached a kind of saturation state. Only at temperatures as high as 700–790 °C some additional increase of the third Sr species was found for sample LSC01. As already shown by the SEM images, on sample LSC01 the exposure to the highest temperatures led to formation of small particles on the LSC surface. These precipitates may be larger accumulates of the third Sr-oxide species or of a newly forming Sr rich phase. However, an unambiguous identification of the particles from XPS spectra is unfortunately not possible from the data available so far. On the second sample (LSC02) even at ca. 700 °C any further changes could be observed, which may be explained by some deviations in true surface temperature between the two samples that can easily arise from a slightly different position of the sample on the sample holder.

**Fig. 7:**
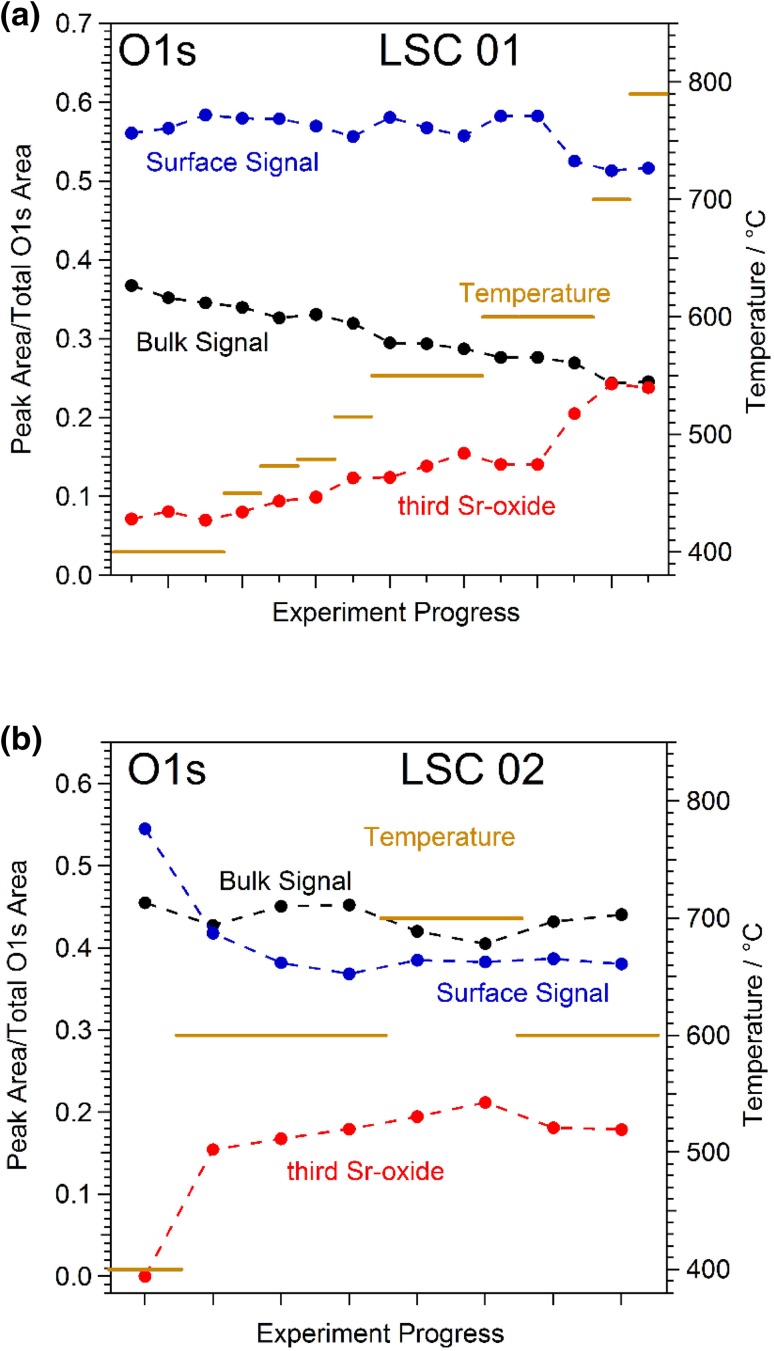
Evolution of the different components of the O 1 s XPS feature with increasing temperature in 0.5 mbar O_2_. The respective peak areas for the perovskite surface (blue), bulk species (black) and the segregated Sr-oxide (red) were obtained from peak fitting of the O 1 s peak. **a** Sample LSC 01. **b** Sample LSC 02

A qualitatively very similar behaviour as for Sr 3d species can also be found for the O 1 s components, which is shown in Fig. 7. Again, the third Sr-oxide species clearly increased upon heating and especially for sample LSC02 a kind of surface saturation can be observed for temperatures of 600 °C and above.

**Fig. 8:**
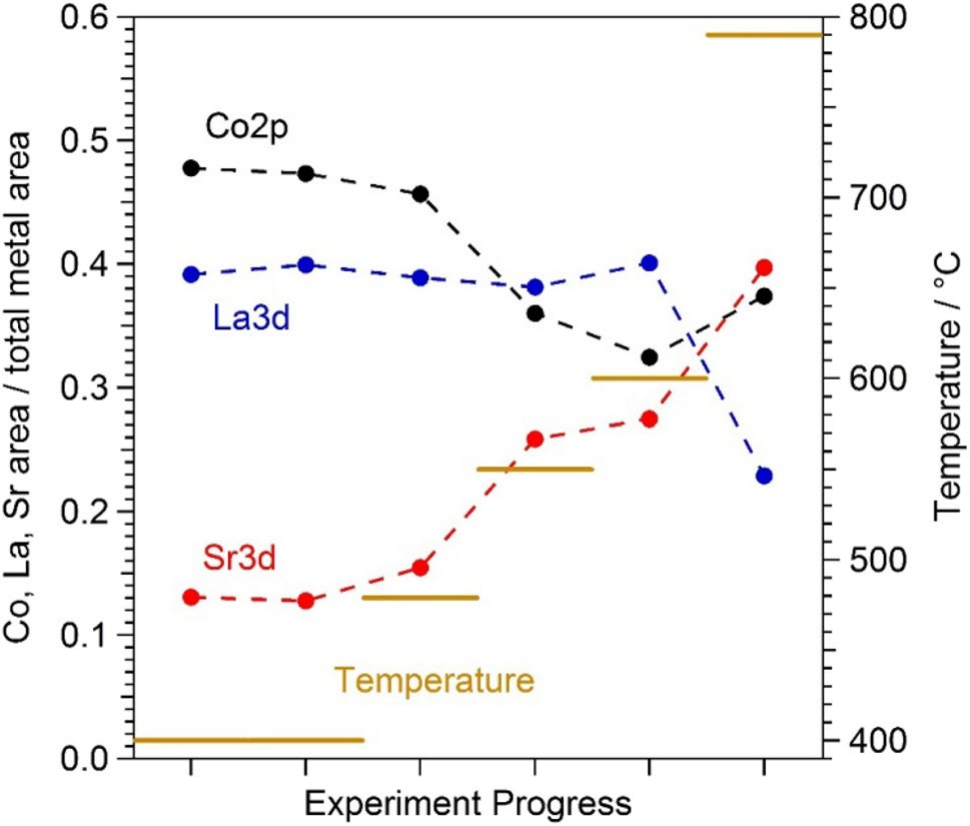
Surface composition of the perovskite electrode (sample LSC01) with increasing reaction temperature at 0.5 mbar O_2_. The respective elemental peak areas (corrected for cross section and photon flux) of the individual components Sr, La, and Co were normalised by the combined cation peak area. The corresponding image for sample LSC02 is shown in the SI (Fig. S7)

Furthermore, it is very interesting that the La signal was virtually unaffected up to 600 °C and only showed a significant drop at the highest investigated temperature, while the Sr signal further increased—see Fig. 8. The first effect will be discussed in more detail below. The latter effect might be related to the formation of presumably Sr-rich particles, which can also be observed for this sample in SEM images (cf. Fig. 5c).

The actually most interesting result is obtained when comparing the XPS results showing the chemical evolution of the LSC surface with the results of simultaneously performed impedance measurements. This electrochemical characterization yielded an initial total polarisation resistance of R_pol,start_ = 308 Ωcm^2^ at ca. 400 °C, which increased within ca. 3.5 h at this temperature to R_pol,end_ = 490 Ωcm^2^. These values are in very good agreement with previously measured resistances under very similar conditions [23]. From the resistance at the beginning and the end of each temperature step an average degradation rate ${\bar{\text{D}}}$ can be calculated by

2 $$\overline{{\text{D}}} =\;\frac{{{\text{R}}_{{{\text{pol,end}}}} - {\text{R}}_{{{\text{pol,start}}}} }}{{{\text{R}}_{{{\text{pol,start}}}} }} \times {\text{100}}\;\% \times \frac{{\text{1}}}{{{\text{t}}_{{{\text{degrad}}}} }}$$

with t_degrad_ denoting the degradation time. From the evolution of the polarisation resistance at ca. 400 °C an average degradation rate ${\bar{\text{D}}}$ ≈ 17%/h results. Since the polarization resistance of the LSC electrode is inversely proportional to the equilibrium exchange rate of Eq. 1 (i.e., the oxygen exchange reaction), this degradation rate is equivalent to a loss of catalytic activity of the LSC surface for O_2_ reduction by 17%/h. The ionic conductivity of the YSZ electrolyte did not significantly change during this period. Thus temperature changes cannot explain the observed electrode activity changes. The obtained value of (8.4 ± 0.4) × 10^−5^ S/cm corresponds to a temperature of 408 ± 2 °C [52, 53], which is in reasonable agreement with the value of ca. 400 °C obtained by the pyrometer.

After increasing the temperature to about 450 °C the polarisation resistance of the LSC01 surface initially decreased to 148 Ωcm^2^ due to the increased temperature, and the thermal activation of the electrode processes. However, at this slightly higher temperature already a significantly faster degradation of the electrode kinetics could be observed. When the temperature was further raised to 479 °C, the polarisation resistance of the perovskite surface increased from 127 to 491 Ωcm^2^ within ca. 45 min corresponding to the enormous average degradation rate of 382%/h. The conductivity of the YSZ electrolyte stayed constant at (4.5 ± 0.2) × 10^−4^ S/cm. From this value a temperature of 475 ± 2 °C can be obtained [52, 53], which again reasonably fits to the value of 479 °C measured by the pyrometer, thus again ruling out any temperature drifts as the reason for the observed polarisation resistance changes.

**Fig. 9:**
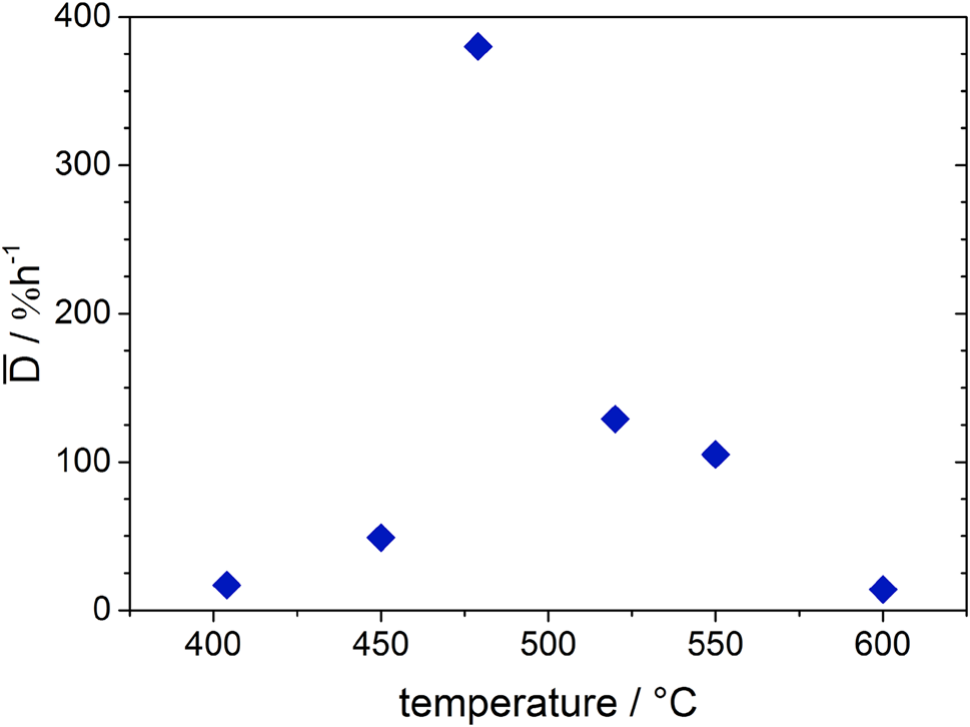
Plot of the average degradation rate of the surface resistance as a function of temperature indicating a distinct maximum between 450 and 500°C

Interestingly, for even higher temperatures the average degradation rate of the polarisation resistance significantly decreased. A plot of ${\bar{\text{D}}}$ versus temperature is depicted in Fig. 9, which shows a distinct maximum between 450 and 500 °C. At temperatures as high as 600 °C the degradation rate again drops to rather low values and like from NAP-XPS data a kind of a saturation effect of the surface degradation can be concluded. A qualitatively comparable trend was found for LSC02.

Comparing this plot with the evolution of the Sr species in Figs. 6 and 7, the highest degradation rate falls into the regime of the steepest increase in the amount of the third Sr species at the surface. In a recent paper employing in-situ deposition of SrO, La_2_O_3_, and Co_3_O_4_ by PLD, cobalt sites—possibly related to surface defects—were suggested as the electrochemically most active sites on the LSC surface [23]. Even small amounts of such sites can be deactivated very effectively by Sr-oxide deposited on the LSC surface. The third Sr species found here might thus act in a very similar manner by covering the electrochemically active Co sites thus lowering the electro-catalytic activity of the LSC surface for the O_2_ reduction reaction (Eq. 1). Indeed, in the present XPS study evidence for this coverage effect can be found. Figure 8 shows the evolution of relative contributions of Sr, La, and Co together with the temperature program. A distinct drop in the cobalt signal occurs exactly after passing through the intermediate temperature region with the highest electrochemical degradation rates. These results strongly support the idea that Sr segregating from the bulk of LSC to its surface decreases its activity for catalysing the electrochemical oxygen exchange reaction by selectively covering the active Co sites. Moreover, these results show that temperatures around 450 °C are high enough for triggering the segregation of sufficient amounts of Sr-oxide to severely degrade the electrochemical activity of LSC electrodes.

### Model of LSC Surface Evolution

With the experimental observations discussed so far, we can suggest a novel model of LSC surface evolution. This model has to consider the following effects:

- A step-like increase in Sr and O surface species upon heating to ca. 400 °C (see Fig. 4).
- Only slow electrochemical degradation at ca. 400 °C with only tiny changes in surface chemistry (cf. Figs. 4, 6).
- Very strong electrochemical degradation at temperatures around 450–550 °C accompanied by the fast formation of a third Sr as well as O species in XPS spectra (see Fig. 6).
- During the electrochemical degradation the intensity of the Co 2p XPS signal decreases while the La 3d signal stays virtually constant (see Fig. 8).
- The surface composition reaches a rather stable condition at temperatures of 600–700 °C, at least on the time scales considered here, while the surface exchange resistance still exhibits a very slow degradation process (cf. Figs. 2c, 6) A very similar behaviour was also observed in a related LEIS study on the surface evolution of LSC [15].
- Above ca. 700 °C the amount of surface Sr detected by XPS starts increasing again, without significantly affecting the surface exchange activity. This change in surface Sr content is accompanied by a decrease of the La 3d signal as shown in Fig. 8. In SEM images the evolution of particles can be observed for this temperature treatment (cf. Fig. 5).

**Fig. 10:**
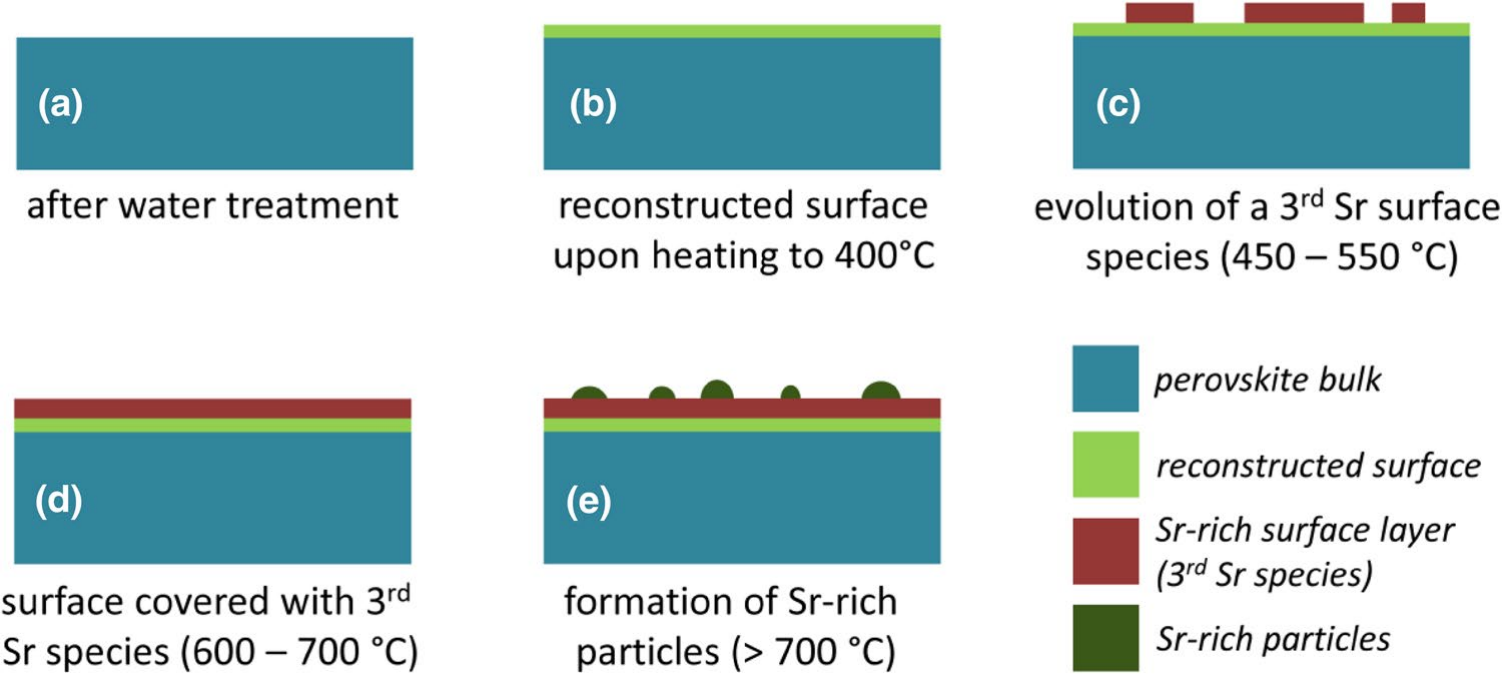
Sketch of the evolution of the LSC surface upon heating in 0.5 mbar O_2_

In the following the observed steps of surface evolution and electro-catalytic activity degradation are discussed—a corresponding sketch is depicted in Fig. 10. Freshly prepared and water-treated LSC (see Fig. 10a) is expected from previous LEIS experiments to exhibit a surface termination with a decreased Co concentration but a Sr/La ratio like in the bulk of the material [15]. Upon heating to ca. 400 °C this surface undergoes some changes—cf. Figure 4 and sketch in Fig. 10b—which can either be related to a surface reconstruction or to decomposition/oxidation of minor foreign surface species such as hydroxyls or adventitious carbon. These surface impurities may have formed either during water treatment or upon the short exposure of the LSC electrode to air between water treatment and introduction into the XPS chamber. After this transformation, the established surface state turns out to be rather stable at ca. 400 °C showing only slow electrochemical degradation at as well as only tiny changes in XPS response.

Heating to higher temperatures, however, triggers formation of an additional Sr-rich species on the surface of LSC (see Fig. 10c), which is accompanied by an increasing deactivation of the electrochemical performance. This distinct correlation of decreasing electro-catalytic activity of the LSC surface with the appearance of the third Sr-oxide suggests this species to be responsible for the observed severe performance degradation of LSC electrodes above 450 °C. Since Co is expected to be the highly active site for the oxygen exchange reaction [23] and since the Co signal decreases while the third Sr species is formed, Co surface sites are concluded to act as the preferential formation spots of the third Sr-oxide species especially during early stages of degradation.

Upon advancing degradation, also larger parts of the surface become covered by the third Sr species and its contribution to the overall Sr 3d signal increases to 35–50% (cf. Fig. 6). Such a formation of a Sr-O rich surface layer is also reported in literature [15, 17–20, 26], but its formation was never observed in-situ so far. In case of temperatures not exceeding 600–700 °C the LSC surface appears to be stabilised in this condition (see Figs. 6, 7 as well as sketch in Fig. 10d). At this stage of degradation, only minor surface chemical changes can be observed by XPS. However, the electrochemical resistance still keeps slowly increasing—see Fig. 6. Actually, this is in accordance with previously published studies, reporting a self-limitation after surface segregation of a certain amount of Sr under moderate temperatures [15, 17]. Also in these experiments the electrochemical degradation was not completed after the amount of segregated surface Sr had virtually stabilised. This behaviour can be understood when assuming that at this metastable state of degradation the third Sr-oxide found in the present study already covered almost all of the active surface Co sites. Since the surface of our LSC electrodes is far from being an ideally flat surface with only one crystallographic orientation, the remaining Co sites may be related to surface defects such as step edges, kinks or grain/domain boundaries. Thus, tiny additional amounts of the third Sr species, presumably below the detection limits of XPS and LEIS, very effectively degenerate these few remaining active spots, thus leading to an ongoing degradation of the electrochemical performance. Actually, this process was heavily accelerated in a previous study by utilising in-situ PLD deposition of SrO on the LSC surface to show that small amounts of surface Sr can lead to strong effects on the electrochemical oxygen exchange resistance [23].

Owing to the fact that the observed strong increase in the third Sr-oxide does lead to a decrease in the Co 2p intensity but not to a significant attenuation of the La 3d signal (see Fig. 8), the new surface species is suggested to exist in form of a La containing Sr-oxide layer. The exact composition of this layer, however, cannot be clarified unambiguously from our data available so far. For a distinct identification of its nature (i.e., composition, crystallography, morphology) additional experiments need to be conducted.

A further increase in Sr content at temperatures exceeding ca. 700 °C is interpreted as the formation of Sr-rich particles on top of the degraded LSC surface (cf. Fig. 10e). This interpretation is supported by the evolution of particles on the electrode surface observable by SEM (cf. Fig. 5) as well as by studies in literature reporting similar effects [19–21, 49]. The particles may be of the same composition as the third Sr-oxide species discussed above. However, since at the highest temperatures also a significant decrease of the La signal was observed (see Fig. 8), the particles may also consist of a regular bulk Sr-oxide phase. Further details on Sr-oxide particle formation on LSC electrodes will be the topic of forthcoming work.

## Conclusions

In this study, the evolution of the polarization resistance of model-type LSC thin film electrodes with increasing temperature was studied by electrochemical impedance spectroscopy while simultaneously performing NAP-XPS experiments. This in-situ characterization allowed a correlation of surface chemical changes and the electro-catalytic activity of LSC for the O_2_ reduction reaction.

At lower temperatures (ca. 400 °C) only slow changes of both surface composition and oxygen exchange resistance can be recorded. A temperature increase to 450–500 °C caused a significant change in the Sr and O chemistry of the LSC surface, accompanied by a severe degradation of the electrochemical activity. From XPS data analysis, formation of an additional Sr-rich oxide phase at the surface is suggested, which was concluded from the appearance of a third species in Sr 3d as well as O 1 s spectra. This third Sr-oxide is discussed to cover electrochemically active Co surface sites, thus very effectively decreasing the electrochemical activity of LSC. Owing to the correlation with the increase in polarization resistance, the formation of this layer can be identified as the responsible process for the first step of degradation of LSC’s catalytic activity. In previous studies this first degradation step was identified as the formation of a SrO monolayer on the surface. From the results of the present study, however, a La and Sr containing oxide layer appears to be a more appropriate description of this Sr-rich surface species.

At higher temperatures (600–700 °C), both the surface chemical changes and the electrochemical degradation became less severe again, indicating the saturation of the surface with the Sr-rich oxide. At temperatures exceeding 700 °C the formation of particles on the LSC surface together with a decrease of the La signal was observed, which did not show a significant effect on the electrochemical performance of LSC.

This identification of the third Sr-oxide on the LSC surface by XPS is a large step towards an understanding of the connection between surface chemistry and electro-catalytic activity of highly active mixed conducting SOFC cathodes. Moreover, these results clearly emphasise the strength of combined electrochemical and in-situ spectroscopic characterization techniques, thus directing the way for future studies striving for an understanding of surface active SOFC cathode materials such as LSC. However, this study also points out the difficulty of unambiguously identifying Sr surface species on Sr-containing perovskites solely from XPS data, which should be regarded as a motivation for a critical analysis of similar spectroscopic data from perovskite-type surfaces.

## Electronic supplementary material

Below is the link to the electronic supplementary material.

Supplementary material 1 (DOCX 1210 KB)
