# Supplementary material for: The Chemical Evolution of the La0.6Sr0.4CoO3−δ Surface Under SOFC Operating Conditions and Its Implications for Electrochemical Oxygen Exchange Activity
Source: Top Catal. 2018 Oct 20;61(20):2129–41. doi: 10.1007/s11244-018-1068-1 (PMC6404788; doi:10.1007/s11244-018-1068-1)
Supplement: Supplementary file 1 — Supplementary material 1 (DOCX 1210 KB) [file 11244_2018_1068_MOESM1_ESM.docx]

Supporting Info to

The chemical evolution of the La_0.6_Sr_0.4_CoO_3-δ_ surface
under SOFC operating conditions and its implications
for electrochemical oxygen exchange activity

Alexander K. Opitz ^a, #^ *, Christoph Rameshan ^b, #^, Markus Kubicek ^a^, Ghislain M. Rupp ^a^,
Andreas Nenning ^a, †^, Thomas Götsch ^c^, Raoul Blume ^d^, Michael Hävecker ^d^,
Axel Knop-Gericke ^d,e^, Günther Rupprechter ^b^, Bernhard Klötzer ^c^, Jürgen Fleig ^a^

a) Vienna University of Technology, Institute of Chemical Technologies and Analytics, Getreidemarkt 9/164-EC, 1060 Vienna, Austria

b) Vienna University of Technology, Institute of Materials Chemistry, Getreidemarkt 9/165-PC, 1060 Vienna, Austria

c) University of Innsbruck, Institute of Physical Chemistry, Innrain 52c, 6020 Innsbruck, Austria

d) Fritz Haber Institute of the Max Planck Society, Department of Inorganic Chemistry, Faradayweg 4­6, 14195 Berlin, Germany

e) Max-Planck-Institute for Chemical Energy Conversion, Dept. of Inorganic Chemistry, Stiftstraße 34-36, 45413 Mülheim, Germany

†) Present Address: MIT, Department of Materials Science and Engineering, 77 Massachusetts Avenue, Cambridge, MA, 02139, U.S.A.

#) authors with equal contribution.

*) corresponding author: alexander.opitz@tuwien.ac.at


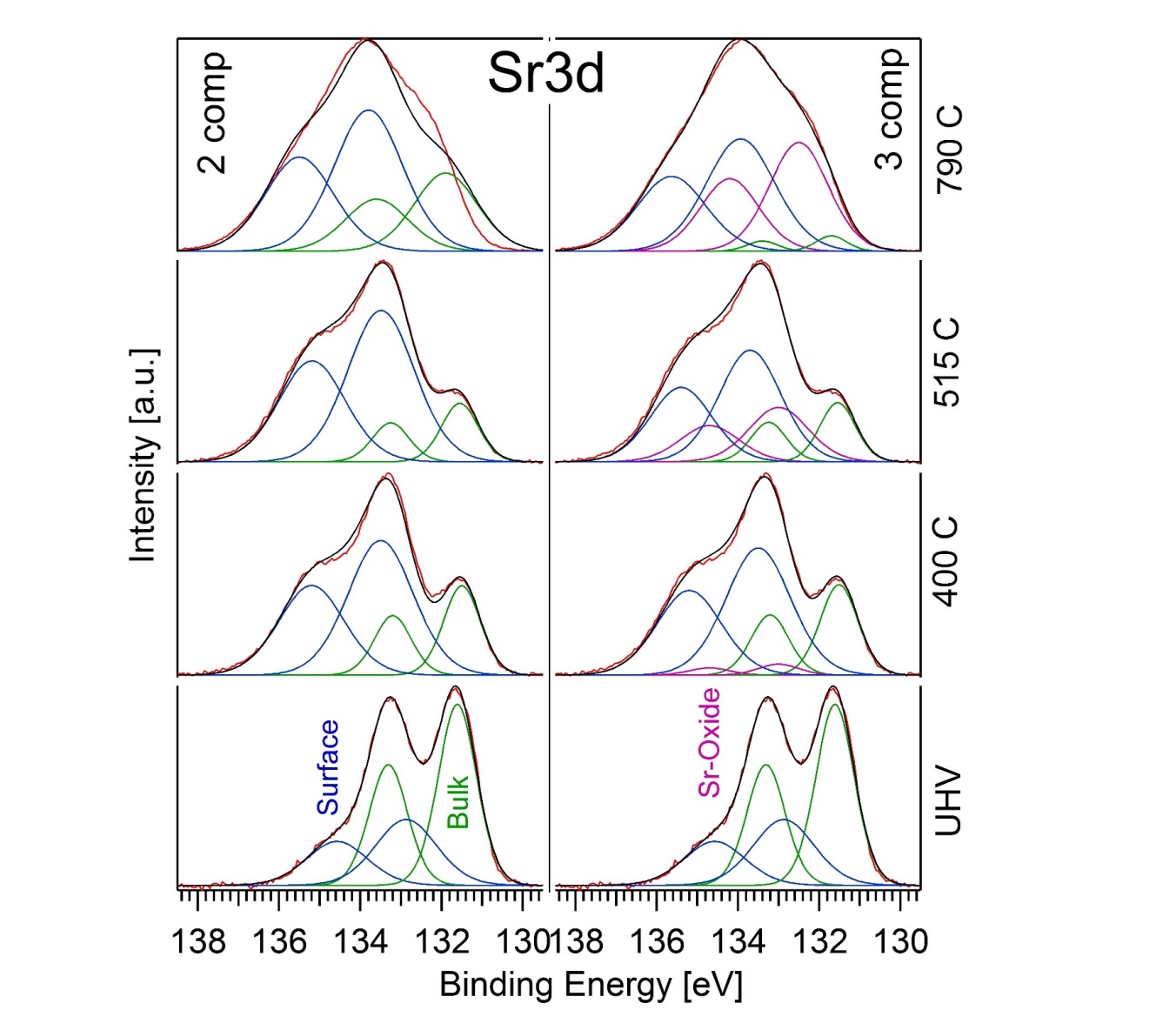


**Figure S1:** Comparison of different fit models for Sr 3d XPS spectra for the virgin, water-treated sample LSC electrode surface directly after introduction into UHV system at room temperature and after heating to 400, 515 and 790°C in 0.5 mbar O_2_. The left panels show fits employing two different species. The right panels show fit results using a three-component-model.


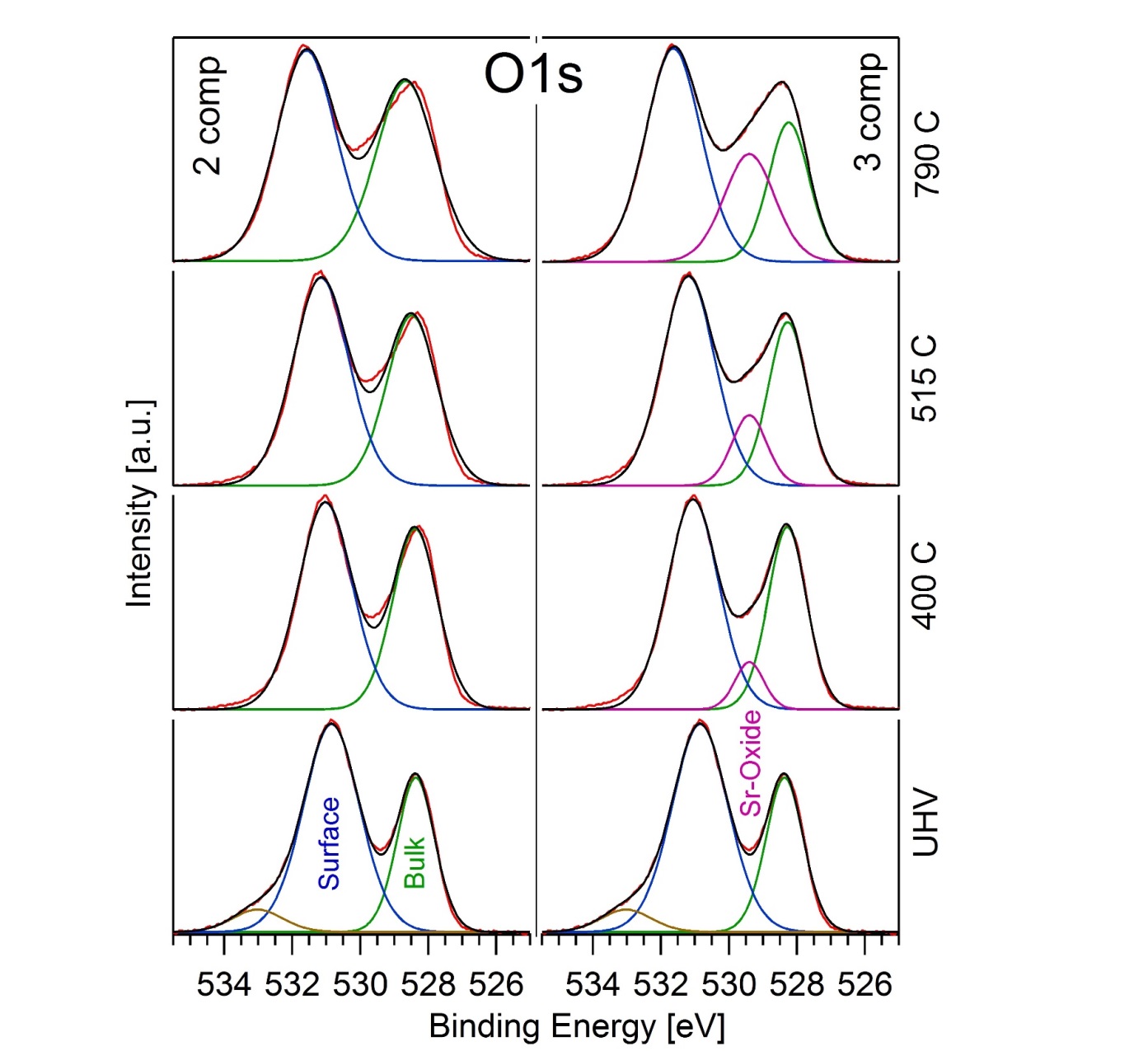


**Figure S2:** Comparison of different fit models for O 1s XPS for the virgin, water-treated sample LSC electrode surface directly after introduction into UHV system at room temperature and after heating to 400, 515 and 790°C in 0.5 mbar O_2_. The left panels show fits employing two different species. The right panels show fit results using a three-component-model.


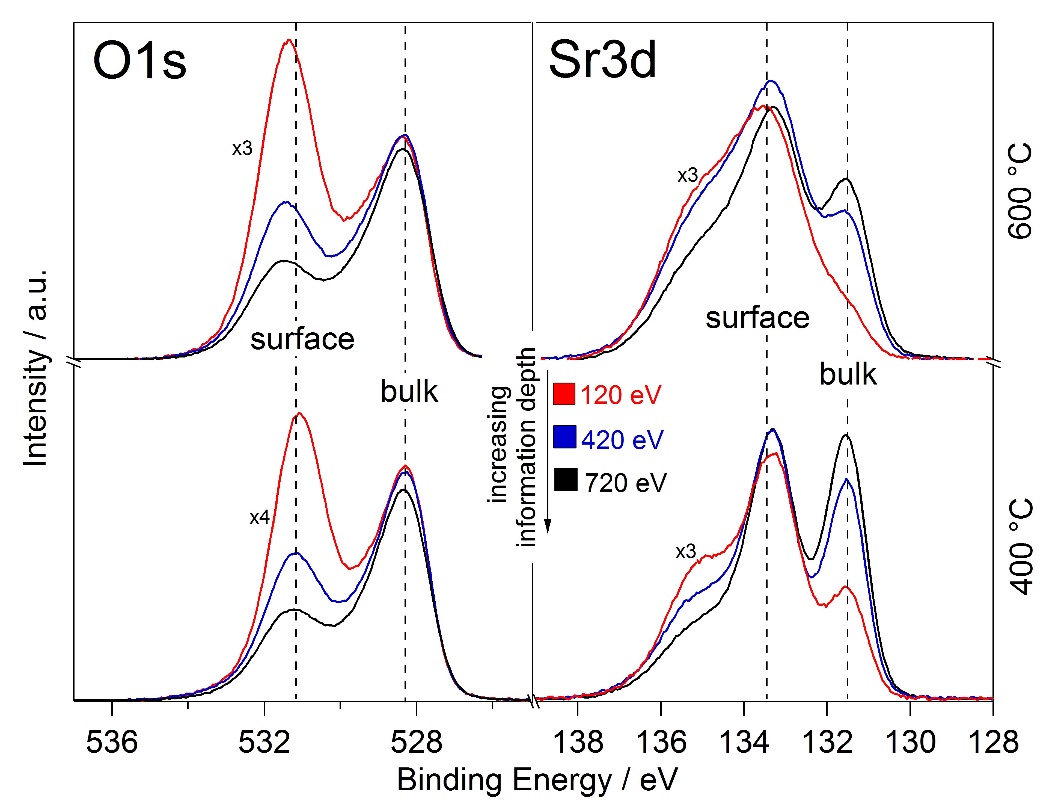


**Figure S3:** Depth profile spectra of the O 1s (left) and Sr 3d (right) signal for ca. 400°C and 600°C measured on sample LSC01 in 0.5 mbar O_2_. The kinetic energy of the photoelectrons was 120, 420 and 720 eV (see legend in the image center). With increasing information depth the intensity ratio surface/bulk decreases for both O 1s and Sr 3d.


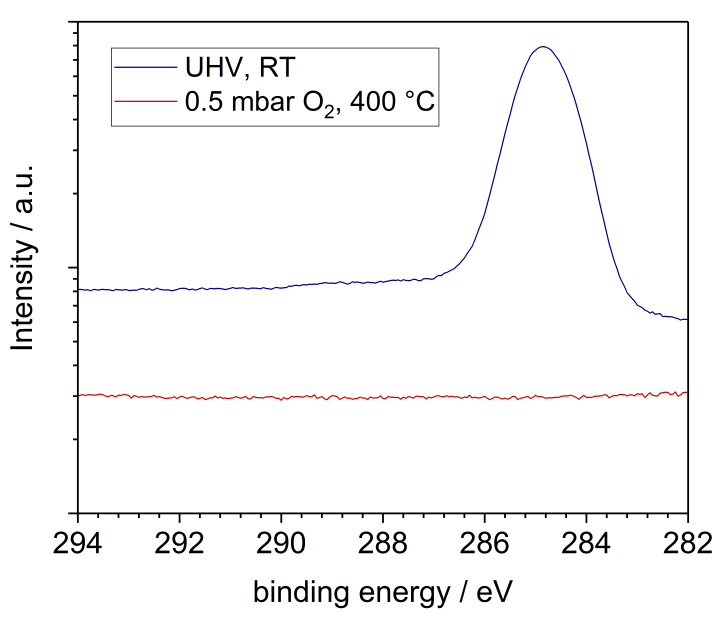


**Figure S4:** C 1s photoelectron spectra recorded on a virgin water-treated LSC sample before any heating (UHV, RT) and after heating to 400 °C in 0.5 mbar oxygen atmosphere. It can be clearly seen that on the virgin sample only adventitious carbon (peak at 284.8 eV binding energy) but not carbonates are present. The carbon was completely removed by heating to 400°C in 0.5 mbar O_2_.


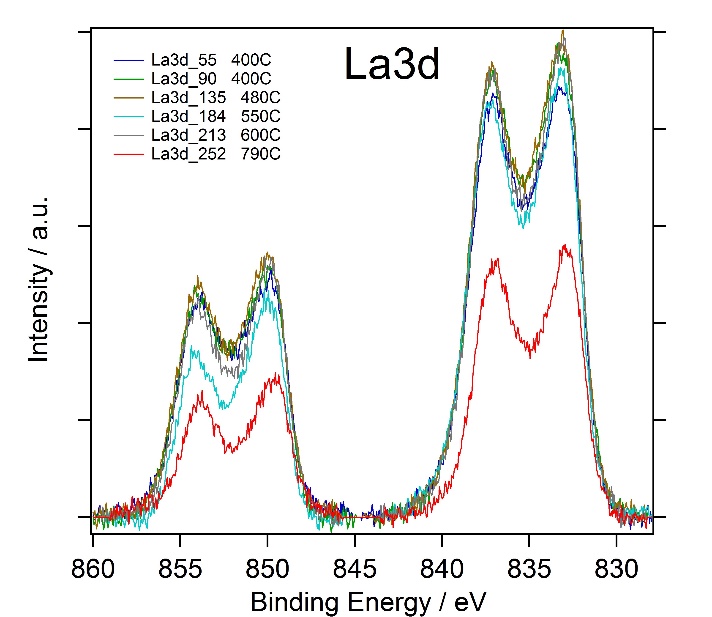

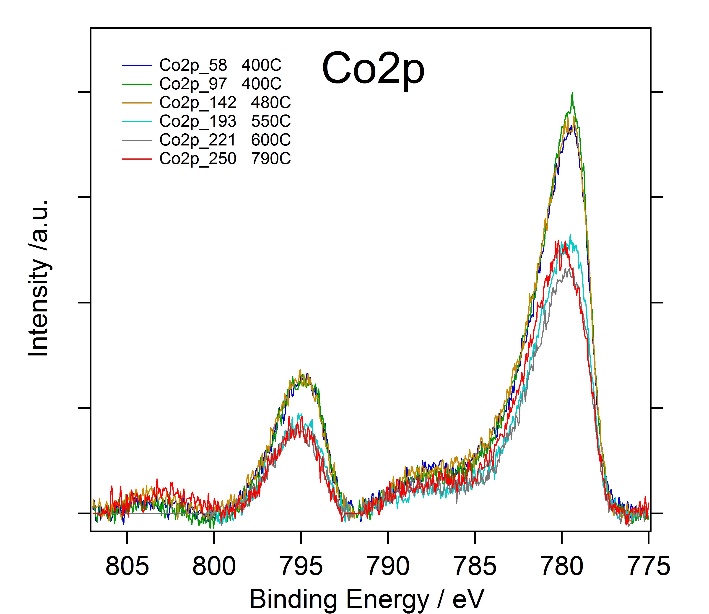


**Figure S5:** Evolution of La 3d (left panel) and Co 2p (right panel) XPS spectra measured on sample LSC01 upon increasing temperature. In case of La only at very high temperatures a decrease can be observed while in case of Co already between 480 and 550 °C a significant decrease of the signal intensity can be observed.


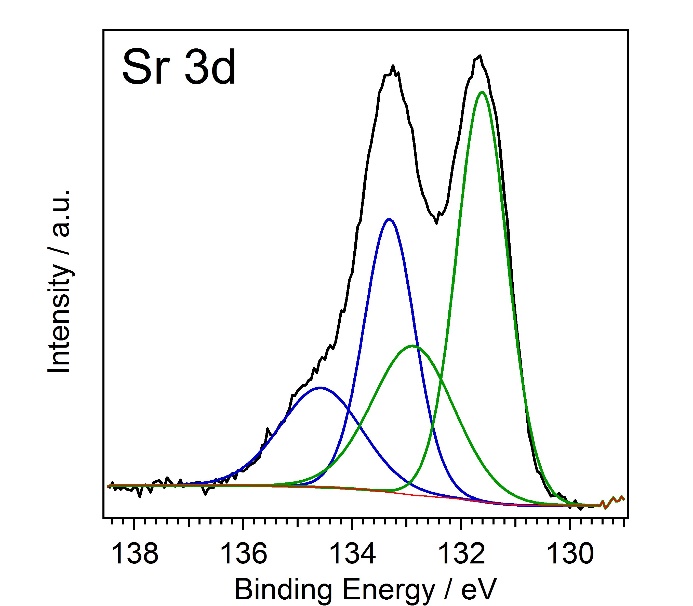

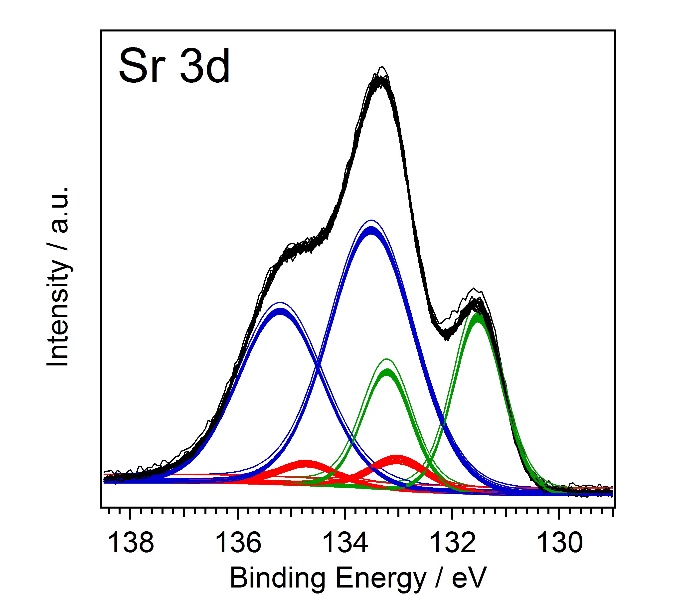


**Figure S6:** Left panel: Initial state of the LSC 01 sample (UHV RT) after introduction into the UHV system. Right panel: All spectra obtained on sample LSC01 at ca. 400 °C in 0.5 mbar O_2_ on the working electrode surface. It shows that the electrode surface does not significantly change during approximetaly 3.5 hours of operation at ca. 400 °C.


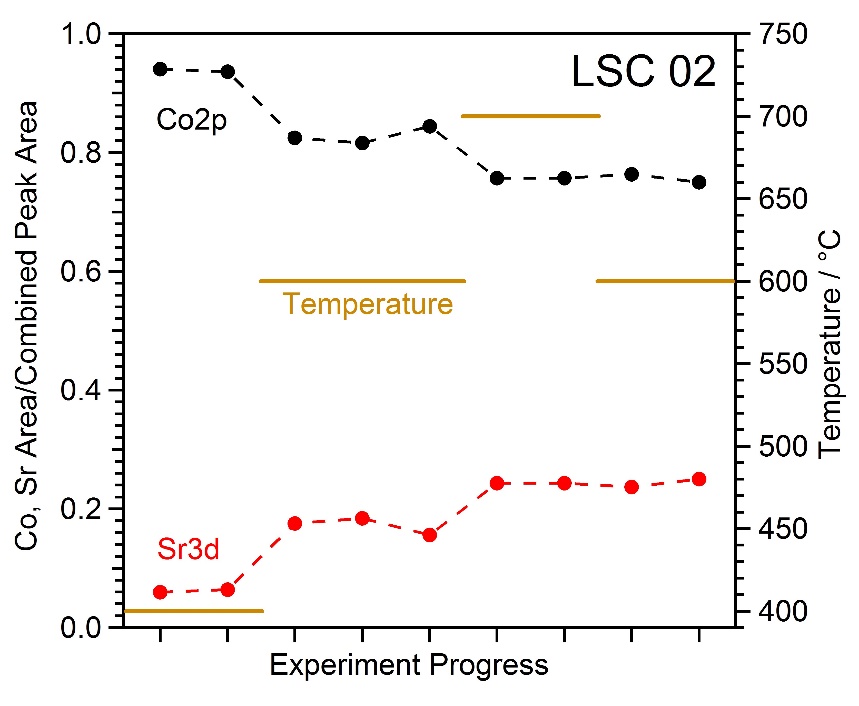


**Figure S7:** Surface composition of the perovskite electrode (sample LSC02) with increasing reaction temperature at 0.5 mbar O_2_. The diagram shows normalized intensities plotted versus measurement number.
